# Si-HgTe Quantum Dot Visible-Infrared Photodetector

**DOI:** 10.3390/nano15040262

**Published:** 2025-02-10

**Authors:** Lei Qian, Xue Zhao, Kenan Zhang, Chen Huo, Yongrui Li, Naiquan Yan, Feng Shi, Xing Peng, Menglu Chen

**Affiliations:** 1 School of Optics and Photonics, Beijing Institute of Technology, Beijing 100081, China; 3220230621@bit.edu.cn (L.Q.); 3120245405@bit.edu.cn (X.Z.); 3120230607@bit.edu.cn (C.H.); lyr@bit.edu.cn (Y.L.); 3220220480@bit.edu.cn (N.Y.); 2 National Key Laboratory of Materials for Integrated Circuits, Shanghai Institute of Microsystem and Information Technology, Chinese Academy of Sciences, Shanghai 200050, China; 3 Zhejiang Key Laboratory of 3D Micro/Nano Fabrication and Characterization, Westlake Institute for Optoelectronics, Hangzhou 311421, China; heqi0508@163.com; 4 National Key Laboratory on Near-Surface Detection, Beijing 100072, China; 5 Laboratory of Science and Technology on Integrated Logistics Support, Changsha 410073, China; shifeng@nudt.edu.cn (F.S.); pengxing22@nudt.edu.cn (X.P.)

**Keywords:** visible-infrared photodetector, HgTe colloidal quantum dot, trans-impedance amplifier circuit

## Abstract

Silicon photodetectors are well developed, with the advantage of their low cost and easy fabrication. However, due to the semiconductor band gap limitation, their detection wavelength is limited in the visible and near-infrared ranges. To broaden the detection wavelength, we stacked a mercury telluride (HgTe) colloidal quantum dot (CQD) photodiode and a silicon PIN photodiode in series. This detector shows response spectra ranging from visible to short-wave infrared (430 nm to 2800 nm) at room temperature. At zero bias, the total photocurrents are 112.5 μA and 1.24 μA, with a tungsten lamp and a blackbody serving as light sources, respectively. The response speed can reach 1.65 μs, with the calculated detectivities of the visible wavelength D* = 1.01 × 10^11^ Jones, and that of the short-wave infrared being D* = 2.66 × 10^10^ Jones at room temperature. At the same time, with a homemade trans-impedance amplifier (TIA) circuit, we demonstrate the device application for figuring out the amplified voltage of the VIS, SWIR, and the VIS-SWIR stacked layers.

## 1. Introduction

Photodetectors have been widely applied in photoelectric imaging, optical communication, and remote sensing [1,2,3,4]. The majority of photodetectors currently on the market are fabricated from inorganic semiconductor materials, including silicon and III–V compound semiconductors, amongst others [5,6,7]. In addition, two-dimensional materials have gradually become important materials for fabricating detectors, such as GaTe [8] and WTe_2_ [9]. Specifically, silicon photodiodes have proved to be very effective in the range of visible wavelengths [10,11], whereas InGaAs, InSb, and HgCdTe are prevalently utilized in infrared photodetectors [12]. However, a single-band photodetector falls short in target discrimination, especially in complex scenarios. Hence, wider-spectral-range detectors are becoming more important.

A feasible way to capture a wider spectral range is through stacking different photonic materials. Taking advantage of solution processing, colloidal quantum dots (CQDs), are compatible with silicon-based photodetectors [12,13]. This has made it possible to achieve stacked photodetectors with easy heterogeneous integration.

Over the years, remarkable progress has been achieved in visible-infrared photodetectors. Stylianos Siontas fabricated Ge QD photodetectors on Si/Ge substrates, capable of detecting 400–1100 nm and 1550 nm wavelengths [14]. A prior study reported a high-quality multifold Ge/Si/Ge CQD stacked heterostructure film (>1 μm) for 850–1560 nm near-infrared photodetection [15]. In 2021, Hwang et al. demonstrated a Ge/MoS_2_ heterojunction detector capable of selectively detecting 406–1550 nm wavelengths in different modes [16]. Perovskites have also found extensive applications in the fabrication of photodetectors [17]. A device incorporating MAPbBr_3_ and MAPbI_3_ as photoactive layers enables bias-switchable responses in the 300–570 nm and 630–800 nm ranges [18]. MAPbI_3_ and Si semiconductors have been successfully integrated to facilitate discrimination between visible (400–800 nm) and near-infrared (800–980 nm) ranges [19]. Additionally, a PVK-Si dual-mode photodetector has been developed for imaging in the 400–1100 nm range [20]. Nevertheless, in the wake of the ceaseless progression of science and technology, the application scenarios for photodetectors are becoming increasingly diverse. The requirements for detector performance are getting more stringent. For photodetectors based on Si/Ge and Ge/Si/Ge, the maximum detectable wavelength is merely 1560 nm. Moreover, the stacked perovskite/Si heterostructures possess a relatively narrow spectral response range, with the widest span being from 400 to 1100 nm. In certain application scenarios demanding longer wavelengths, HgTe CQDs have shown promise. HgTe CQDs have been developed rapidly in recent years, with photodetectors capable of covering the short-wave infrared to even very long-wave infrared ranges [21,22,23,24]. On the other hand, the size distribution of HgTe CQDs can be effectively narrowed from 12% to 6%, along with enhanced crystallinity and sharper absorption features [25]. Furthermore, HgTe CQDs have been demonstrated to be applicable in fabricating detectors with excellent performance [26,27]. Xue et al. developed a high-operating-temperature mid-infrared photodetector with a QD gradient homojunction. The detector exhibits a responsivity of 2.7 A/W and a quantum efficiency exceeding 77% [26].

For the realization of multi-band light detection, the stacked multijunction structure represents a favorable alternative, which has been employed in multijunction solar cell technology. For example, Geisz et al. reported a six-junction inverted metamorphic structure (6J IMM) that was grown on a GaAs substrate [28]. Laoufi et al. simulated a double-junction CGS/CIGS solar cell structure, which encompassed a ZnO window layer, a CdS buffer layer, CGS and CIGS absorber layers, as well as a Mo back electrode [29].

In this work, we demonstrate a visible-infrared photodetector through the vertical stacking of HgTe CQD and silicon. The HgTe CQD photodiode is based on a PN junction covering wavelengths of up to 2800 nm with a detectivity of 1.41 × 10^10^ Jones. Its response speed can reach 1.65 μs. The silicon photodiode is based on a PIN junction covering 430 nm to 1100 nm wavelengths with a specific detectivity of 6.76 × 10^10^ Jones. Its response speed is merely 0.59 μs. The visible-shortwave infrared (VIS-SWIR) stacked photodetector can cover 430 to 2800 nm wavelengths. At room temperature, the calculated detectivities are D* = 1.01 × 10^11^ Jones for the visible layer, and D* = 2.66 × 10^10^ Jones for the short-wave infrared layer. Furthermore, we design a trans-impedance amplifier (TIA) circuit specifically for the aforementioned stacked photodetector to ensure the effective amplification and conditioning of the electrical signals [30]. The photocurrents of 120 μA generated by the stacked photodetector can be amplified to 3 V with an amplification factor of 25,000. The response time of the integrated prototype is 3.84 μs. The amplification factor can be adjustable within the range of 100 to 200,000 times, which is sufficiently fast to probe multiple fields.

## 2. Materials and Methods

### 2.1. Synthesis of HgTe CQDs

The precursor solution was prepared with 0.1 mmol of HgCl_2_ (mercuric chloride) powder dissolved in 4 mL OAM (oleylamine). In the nitrogen environment, it was stirred at 105 °C for about 1.5 h. Meanwhile, 0.3 mL TOP (tri-n-octyl-phosphine) and 0.1 mL DDT (dodecyl mercaptan) were combined with 4 mL of TCE (tetrachloroethylene) to prepare a quenching solution. The TOPTe solution was prepared by using 0.1 mmol Te (tellurium) and 0.1 mL TOP [27]. Waiting for the precursor solution to become clear, it was cooled to 80 °C. Following this, 0.1 mL TOPTe was injected swiftly, with the observation that the solution turned black at once. After 4 min, the quenching solution was quickly poured into the above solution to terminate the reaction. To stop the final reaction and cool the solution to room temperature, a water bath was necessary. In the last step, 1.5 mL DDAB (dimethyldioctadecylammonium bromide) and 40 mL IPA (isopropyl alcohol) were added to the raw reaction solution of HgTe CQDs and centrifuged. The precipitate was filtered and dissolved in 0.25 mL CBZ (chlorobenzene) to obtain short-wave infrared HgTe CQDs.

### 2.2. Synthesis of Ag_2_Te CQDs

In the nitrogen glove box, 0.2 mmol AgNO_3_ (Argentum nitricum) was dissolved in 5 mL OAM and 0.5 mL OA (oleic acid), adjusting the temperature to 70 °C and stirring the solution for 30 min. Subsequently, 0.5 mL of TOP was injected into the solution at 160 °C, and the solution turned yellow after 40 min of reaction. Then, 0.1 mL of TOPTe was added to the solution. After 10 min, the solution was removed from the glove box to cool and clean. The Ag_2_Te CQDs can be obtained. At last, the Ag_2_Te CQDs were dissolved in 1:9 hexane:octane solution for device preparation.

### 2.3. Fabrication of VIS-SWIR Stacked Photodetector

A 50 nm ITO (Indium Tin Oxide) film was deposited on the substrate of the visible photodetector, a silicon PIN diode chip, by magnetron sputtering. After that, the substrate was successively placed into deionized water, IPA, and acetone, cleaning for 5 to 10 min by ultrasonication. Subsequently, it was carefully picked out with tweezers and dried with a nitrogen gun. Importantly, the chip was supposed to be treated with MPTS (3-mercaptopropyltrimethoxysilane) for 30 s to strengthen the adhesion between the substrate and the nanoparticle solution. MPTS can enhance the adhesion of quantum dots on the surface through chemical bonding [31], and it is frequently utilized as an adhesive in chemical synthesis [32].

The HgTe CQDs were deposited on the substrate of the treated chip, using the spin-coating method to form an HgTe CQD film. The film was placed into the HgCl_2_/MeOH and EDT (ethanedithiol) solution one after another, with each immersion lasting for 10 s. The deposition was stopped when the film thickness reached approximately 400 nm. The Ag_2_Te CQDs were deposited on the upper layer of the HgTe CQD film following the same steps until the thickness reached approximately 20 nm. Finally, 50 nm of Au film was deposited as the top electrode using evaporation coating.

### 2.4. Design of TIA Circuit

Considering the feeble output current of the detector, a TIA circuit was meticulously engineered to amplify the tiny current generated by the specific detector. The dark current originating from the detector could be nullified by an offset circuit composed of ±5 V direct current (DC) voltage sources and a 1 MΩ adjustable resistor. Concerning the amplification segment, the OPA657U was selected as the core operational amplifier. This amplifier incorporated high-gain bandwidth, low distortion, and a low-voltage noise junction field-effect transistor (JFET) input stage. This design endowed a substantial dynamic range for the driving of a high-precision analog-to-digital converter (ADC). Incorporating capacitors into feedback circuits can effectively reduce circuit noise and adjust bandwidth, so a 30 pF capacitor was chosen. Since a single large resistance will augment the instability of the circuit, a 100 Ω resistor and a 200 kΩ variable resistor were employed as feedback resistors. To suppress the noise and spurious waves in the circuit, a filter circuit and a voltage stabilization module were devised.

### 2.5. Characterizations

Spectral response: the spectral response of HgTe CQD film was measured by a Nicolet iS20 FTIR (Tampa, FL, USA) [22]. The spectral response of visible light was measured by N4S UV-VIS spectrophotometer (INESA, Shanghai, China).

I–V Curves: photocurrent and dark current were measured by a Keithley 2602B source meter (Shanghai Yunjian Intelligent Technology, Shanghai, China), with an HT-P1000 blackbody source at 600 °C and a tungsten lamp.

Response speed: the optical response speed of the dual-band detector was measured by the DG2102 oscilloscope (Tektronix, Beaverton, OR, USA), where modulated visible and 1550 nm infrared lasers are used as light sources, respectively.

Film thickness: film thickness was measured by an Alpha-Step D-300 stylus profiler (KLA-Tencor Corporation, Milpitas, CA, USA).

Circuit characteristics: voltage, resistance, and current were tested by multimeters (ZTY890D, Zhejiang Zhengtai Instrument Co., Wenzhou, Zhejiang, China).

Simulation: Multisim and Proteus were used for circuit construction and simulation.

## 3. Results and Discussion

### 3.1. Characterizations of Single-Band Photodetectors

**Figure 1:**
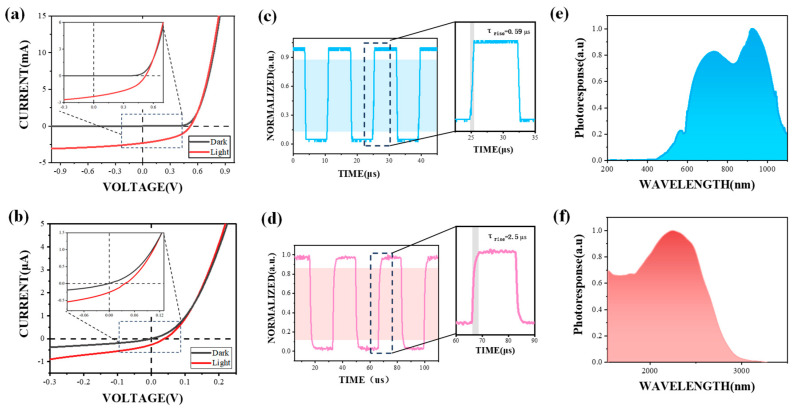
Characterization of photodetectors. (**a**,**b**) I–V curve characterization on the VIS photodetector and the SWIR photodetector with a blackbody at 600 °C and a tungsten lamp as light sources, respectively. (**c**,**d**) The response speed of the VIS photodetector and the SWIR photodetector measured with the incident light modulated at 70 kHz and 30 kHz, respectively. The red and blue parts in the background represent the regions where the rise time of the VIS and the SWIR photodetector increases from 10% to 90%, respectively. The light sources are a visible laser and a 1550 nm infrared laser, for VIS and SWIR photodetectors, respectively. (**e**,**f**) Response spectra of the VIS photodetector and the SWIR photodetector.

The fabricated silicon PIN photodetector exhibits high sensitivity in the visible (VIS) band, and the HgTe CQD photodetector demonstrates sensitivity in the short-wave infrared (SWIR) band. The characterizations of the VIS and SWIR photodetectors are shown in Figure 1, respectively. When a tungsten lamp is employed as the light source, the photocurrent of the VIS photodetector reaches 2.27 mA at a distance of 20 cm from the sensitive surface of the chip (Figure 1a). The response speed, defined as the rising time constant (τ_rise_), refers to the time needed for the signal voltage to rise from 10% of the maximum value to 90% [22]. As depicted in Figure 1c, when a modulated visible laser is employed as the light source, the τ_rise_ of the VIS photodetector is 0.59 μs, and the time constant decay (τ_decay_) is approximately 0.39 μs. Furthermore, the spectral response range of the VIS photodetector (Figure 1c) is 430–1100 nm with a peak wavelength at 940 nm, covering the visible band and partial near-infrared band.

The I–V curves of the SWIR photodetector are illustrated in Figure 1d. The testing was conducted with a blackbody at 600 °C serving as the light source. Under a voltage of 0 V, the photocurrent reaches 300 nA. When irradiated by a modulated 1550 nm infrared laser, as shown in Figure 1e, the τ_rise_ of the SWIR photodetector is 2.5 μs, and the τ_decay_ is 0.39 μs. The spectrum of the SWIR photodetector (Figure 1f) has a peak at 2500 nm and encompasses the short-wave infrared region, extending up to 2800 nm.

The detectivity is also a preferred value to characterize the photodetector’s performance; it can be described as follows:

(1) $$D^{*}=\frac{\sqrt{A}}{I_{in}}R$$

where A is the area of the detector, $I_{in}$ is the root mean square (RMS) current noise within a bandwidth of 1 Hz, and R is the responsivity. $R=I_{ph}/P$, where $I_{ph}$ is the photocurrent of the detector, and P is the optical power. The active areas of the VIS and SWIR photodetectors are 1.5 mm × 1.5 mm and 0.2 mm × 0.2 mm, respectively. When a blackbody is employed as the radiation source for the SWIR photodetector, the radiation power density of its photoresponse spectrum is 676.135 W/m^2^. Through calculation, the responsivity R is 0.24 A/W. For the VIS photodetector, a tungsten lamp serves as the radiation source; R is 0.73 A/W. The Johnson noise is calculated by $\sqrt{4k_{b}T/r_{dark}}$ = 0.281 pAHz^−1/2^ for the SWIR photodetector, and 1.58 pAHz^−1/2^ for the VIS photodetector. In the aforementioned equation, $k_{b}$ represents the Boltzmann constant, which is 1.38 × 10^−23^ J/K, T = 300 K, and $r_{dark}$ is the resistance linearly fitted by dark current. The shot noise for the SWIR photodetector is calculated by $\sqrt{2eI_{dark}∆f}$ = 0.06 pAHz^−1/2^, and 0.04 pAHz^−1/2^ for the VIS photodetector. Here, e is the elementary charge 1.6 × 10^−19^ C, $I_{dark}$ is the dark current, and ∆f is the bandwidth (1 Hz). The specific detectivities are 1.41 × 10^10^ Jones for the SWIR photodetector and 6.76 × 10^10^ Jones for the VIS photodetector.

### 3.2. Characterizations of VIS-SWIR Stacked Photodetectors

**Figure 2:**
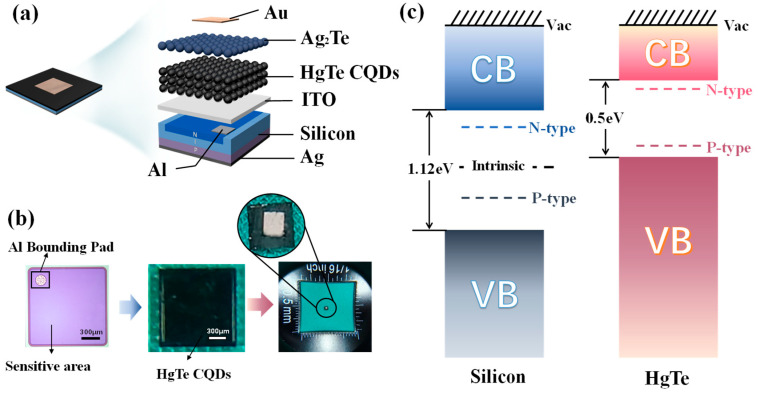
VIS-SWIR stacked photodetector. (**a**) Structure diagram of VIS-SWIR stacked photodetector. (**b**) Real picture of VIS-SWIR stacked photodetector. (**c**) Energy diagram of VIS-SWIR stacked photodetector.

To fabricate the VIS-SWIR stacked photodetector, the HgTe CQD photodetector is stacked onto the VIS photodetector, which is coated with a magnetron-sputtered ITO conductive film. The structure of the photodetector is vertically stacked (Figure 2a). The bottom section of the photodetector comprises a silicon PIN visible photodiode chip, and the entire bottom layer is fully covered with Ag as an anode. The Al electrode on the top of the VIS photodetector is connected to the HgTe CQD photodetector via an ITO film. HgTe CQDs are employed as the sensing material for the infrared band. Ag_2_Te CQD is selected as the doping material. Once Ag_2_Te CQD is deposited onto the HgTe CQD film, the Ag⁺ ions will diffuse into the HgTe CQDs, thereby giving rise to a “p-type” layer [33]. Furthermore, after HgCl_2_ solution treatment, a large number of Ag⁺ ions are released from Ag_2_Te CQD and form AgCl salt. Subsequently, AgCl, which has the property of insolubility, is fixed on the nearest HgTe CQDs [34]. Thus, stable doping in the spatial dimension is achieved. Then, a 50 nm thick Au film, which acts as the cathode, and the underlying Ag electrode serve as the bonding pads for the stacked photodetector.

The purple square chip on the left side (Figure 2b) is the VIS photodetector chip, which has a side length of 1500 μm. The purple area is the sensitive region, while the white section located in the upper-left corner is the Al electrode. Following the deposition of HgTe CQD film, the surface of the chip assumes a blackish-brown hue.

The n–p–n configuration is designed for the VIS-SWIR stacked photodetector. In this device, the VIS and SWIR photodetectors are “p-i-n-type” structures and “p-n-type” structures, respectively. The valence band of silicon is at −5.17 eV and the conduction band is at −4.05 eV at 300 K [35,36]. Given that the band gap width of silicon is 1.12 eV, the intrinsic region consists of a high-purity semiconductor material with a work function of 4.85 eV (Figure 2c). To form the “p-type” layer, doping with trivalent elements is necessary to generate more holes. The work function of “p-type” silicon is 4.95 eV. To create the “n-type” layer, a pentavalent element is doped in silicon. The work function of “n-type” silicon is 4.6 eV. Prior research has indicated that Ag_2_Te nanocrystals were effective “p-type” dopants for HgTe CQDs [34,37]. For 2.5 μm HgTe CQDs, the valence band is at −4.77 eV and the conduction band is at −4.27 eV [38]. The band gap of HgTe CQDs is 0.5 eV, which is relatively narrow. Consequently, the energy needed for electrons to transition from the valence band to the conduction band is low. The HgTe CQD photodetector can absorb photons with lower energy, thereby achieving the infrared response.

**Figure 3:**
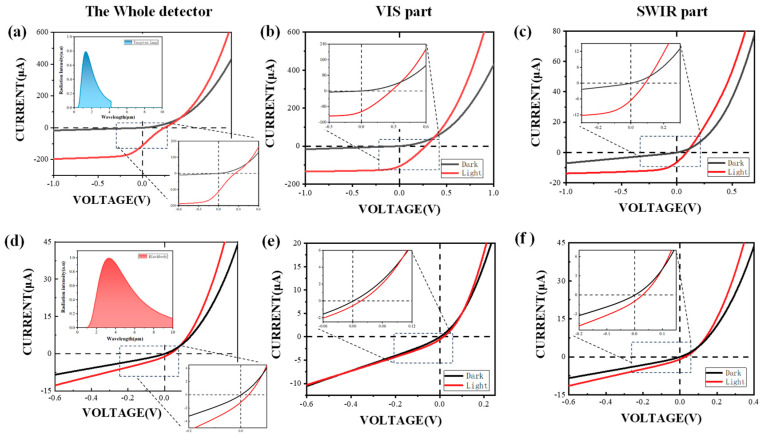
I–V curves of the VIS-SWIR stacked photodetector. I–V curves of light and dark currents between different layers with a tungsten lamp used as the light source. They correspond to VIS-SWIR stacked (**a**), VIS (**b**), and SWIR (**c**) layers, respectively. I–V curves of light and dark currents between different layers with the blackbody at 600 °C used as the light source. They correspond to VIS-SWIR stacked (**d**), VIS (**e**), and SWIR (**f**) layers, respectively. The inserted graph shows the radiation spectra of the tungsten lamp (**a**), and the blackbody (**d**).

To explore whether the photodetector is capable of responding to visible and infrared light, we conduct tests on the photocurrents of the VIS layer, SWIR layer, and VIS-SWIR layer of the photodetector. The I–V curves among different layers of the stacked photodetector are measured. With the tungsten lamp and blackbody serving as light sources, the I–V curves of the VIS-SWIR stacked photodetector layer, VIS layer, and SWIR layer are shown in Figure 3. The tungsten filament lamp encompasses a wider variety of visible wavelengths. On the other hand, at 873 K, the blackbody primarily supplies illumination within the infrared region. When irradiated by these two distinct light sources separately, the stacked detector’s capacity to distinguish between visible and infrared light can be more effectively demonstrated.

When the VIS-SWIR stacked photodetector is irradiated by the tungsten lamp, it can be observed that the I–V curve of the VIS-SWIR photodetector exhibits an S-shape. This result indicates that the junction between the Si PIN photodiode and the CQD photodiode may act as a reverse diode. This drawback could be fixed by adding a buffer layer. For example, Xu et al. utilized SCAPS to simulate the influence of the doping density of the p-type PbS layer and the defect density at the Si/PbS interface on the EQE value, where the inserted buffer layer suppresses interfacial recombination [39]. We consider depositing an additional layer of silver nanoparticles on the ITO in the future to optimize the interface barrier and enhance the conductivity. Moreover, exploring the approach of reducing the doping density in the p-type HgTe layer could also potentially improve the performance of the VIS-SWIR photodetector.

Under the irradiation of the tungsten lamp, the VIS-SWIR stacked photodetector can generate a photocurrent of 112.5 μA (Figure 3a). The photocurrent of the VIS layers is 105.7 μA, and the photocurrent of the SWIR layers is 6.8 μA (Figure 3b,c). The photocurrent generated by the VIS layer is 15.5 times larger than that generated by the SWIR layer since the light intensity of the visible part is relatively higher than that of the infrared part under the tungsten filament lamp.

When the 600 °C blackbody is employed as the light source, the photocurrent of the VIS-SWIR stacked photodetector is 1.24 μA (Figure 3d). Under the same conditions, the photocurrent of the VIS layer is 0.36 μA, which is 293.58 times smaller than the scenario where the light source is a tungsten lamp. The photocurrent of the SWIR layer is 0.88 μA (Figure 3e,f), 2.93 times larger than that of the single-band SWIR photodetector.

For the VIS layer irradiated by the tungsten lamp, $I_{in}$ includes Johnson noise (0.23 pAHz^−1/2^) and shot noise (0.13 pAHz^−1/2^). The D* of the VIS layer is 1.01 × 10^11^ Jones. In the case of the SWIR layer with the blackbody as the light source, P is calculated by $A_{BB}A_{p}\emptyset /L^{2}$ = 5.3 μW/mm^2^, where $A_{BB}$ is the area of the blackbody radiation port (400π mm^2^), $A_{p}$ is the area of the photodetector, ∅ is the luminous flux (676.135 μW), and L is the distance between the blackbody and the photodetector. As R is 0.17 μA/μW and $I_{in}$ is 0.32 pAHz^−1/2^, D* can be calculated to be 2.66 × 10^10^ Jones. The D* of the VIS layer is 1.5 times better than the single-band VIS photodetector, and the D* of the SWIR layer is 1.8 times higher than the single-band SWIR photodetector. This phenomenon demonstrates that the VIS-SWIR stacked photodetector possesses an exceptionally high level of response sensitivity and can react to both visible and infrared bands. Furthermore, this stacked photodetector, in contrast to single-band detectors, can effectively distinguish objects with different color temperatures.

However, the current VIS-SWIR stacked photodetector still encounters several challenges. Firstly, by comparing the photocurrents in the VIS layer of Figure 1a and Figure 3b, it is readily observable that an amount of visible light will not reach the Si PIN photodiode due to the HgTe CQD absorption. This problem could be solved by reversely stacking CQD and silicon. Unfortunately, in this case, the transmittance of the silver metal junction at the bottom layer in the structure is less than 3% due to the thickness.

### 3.3. Trans-Impedance Amplifier Circuit

**Figure 4:**
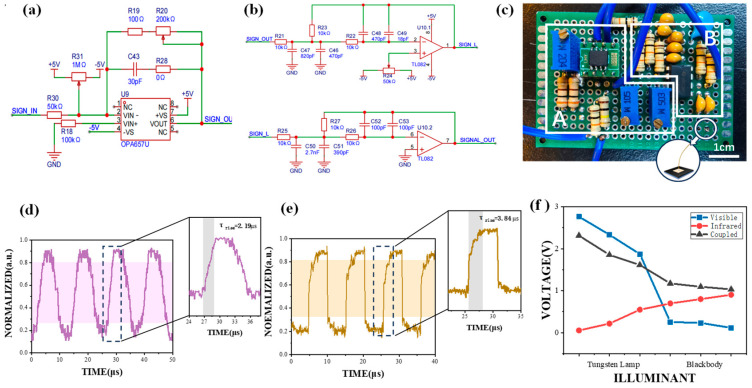
The design and test results of the trans-impedance amplifier circuit. (**a**) A schematic diagram of the cross-resistance amplifier circuit. (**b**) Schematic diagrams of filter and voltage-regulator circuits. (**c**) A real image of the circuit. Part A is the TIA circuit, Part B is the filter and voltage regulator module. (**d**,**e**) The response speed of the TIA circuit and the TIA circuit connected with the VIS-SWIR stacked photodetector with a tunable laser as the light source. The purple and brown parts in the background represent the regions where the rise time of the TIA circuit and the TIA circuit connected with the VIS-SWIR stacked photodetector increases from 10% to 90%, respectively. (**f**) Output voltages of the VIS photodetector (blue), SWIR photodetector (red), and the (black) VIS-SWIR stacked photodetector amplified by the TIA circuit with a tungsten lamp and a blackbody as light sources. The abscissa represents the process in which the photodetector gradually moves away from the tungsten filament lamp and then gradually approaches the blackbody.

The VIS-SWIR stacked photodetector produces a weak current signal as its output, at the magnitude of μA. Direct processing of these signals may be severely interfered with by noise and hard to measure accurately. Converting it to a voltage signal via a TIA circuit is essential for subsequent processing, as voltage signals are more convenient for electronic systems and acquisition devices. Since the current signals from our photodetector are so small (with a maximum of 112.5 μA), much additional research and selection is needed when purchasing an off-the-shelf TIA on the market. So, we have designed a TIA circuit for easier usage and data processing by ourselves. Its schematic clearly displays the process, results, and key design details. In this circuit, amplification can be achieved simply by using a simple feedback resistor, which is more straightforward compared to complex designs such as the T-type feedback network. An offset circuit at the front end of the circuit is specifically designed to counteract the dark current (0–10 μA) of the detector. The current compensating range of this circuit can exceed 1 mA, a value significantly larger than the maximum dark current generated by the photodetector. This ensures that the output voltage of the TIA circuit (Figure 4a) can start at zero, providing a stable and accurate baseline for subsequent signal processing. Subsequently, the filter- and voltage-regulating circuits (Figure 4b) are incorporated after the TIA circuit to stabilize the output voltage and reduce interference noise and extraneous waves. As shown in Figure 4c, the whole circuit is integrated into a compact 4 × 6 cm^2^ Printed Circuit Board (PCB). The PCB is thoughtfully divided into two parts: Part A houses the TIA circuit, while Part B comprises the filter and voltage regulator module. The stacked photodetector’s output pins are linked to the corresponding input pins of the TIA circuit, ensuring a smooth and efficient signal transfer for optimal performance.

An oscilloscope is employed to measure the response speed of the circuit and the coupled photodetector, as shown in Figure 4d,e. In Figure 4d, the signal generated by the function generator is directly fed into the input terminal of the circuit without incorporating any detector. The clear square wave shows that the circuit has a high sensitivity and fast response speed, and is thereby able to meet the requirements of the application. When the output current of the VIS-SWIR photodetector is utilized as the input signal for the TIA circuit (Figure 4e), the measured response speed is 3.84 μs at a frequency of 30 kHz. It can be calculated that the τ_rise_ of the VIS-SWIR photodetector is 1.65 μs. To elucidate whether variations in light intensity could exert an influence on the response speed, we methodically decreased the light intensity to a tenth of its initial level. Subsequently, we observed that there was no discernible alteration in the speed.

The VIS-SWIR stacked photodetector is illuminated by a tungsten lamp first and a blackbody later. The amplification resistors are configured in series, consisting of 100 Ω and 200 kΩ adjustable resistors. This configuration enables the adjustable amplification factor to range from 100 to 200,000 times. The circuit saturation voltage is measured to be 3.6 V, and the maximum photocurrent that the detector can effectively receive is approximately 120 μA. To avoid an inaccurate result stemming from voltage saturation, the amplification factor is set to 25,000 times through the adjustable resistor. The voltage value after amplification can be calculated by Ohm’s law; it can be described as

(2) $$U_{out}=-I_{in}R_{f}$$

where $I_{in}$ is the input current, and $R_{f}$ is the feedback resistance.

Figure 4f displays the output voltage following the circuit. The photodetector is initially illuminated by a tungsten filament lamp. The distance between the tungsten lamp and the photodetector is gradually increased, starting from 20 cm. Subsequently, the photodetector is exposed to a blackbody, and the distance between the blackbody and the photodetector is gradually reduced from a far position to 20 cm. As the distance from the tungsten lamp increases and the distance from the blackbody decreases, the output voltage of the VIS layer exhibits a decreasing trend, whereas the SWIR layer shows an increasing trend. When the stacked photodetector generates photocurrents of 92.4 μA, 74.12 μA, and 43.76 μA, the corresponding amplified voltages are 2.312 V, 1.853 V, and 1.094 V, respectively. The result shows a linear correlation. The output voltage follows the predicted rule, and the measured amplification factor remains stable at 25,000 times.

Finally, we carried out a meticulous comparison between our VIS-SWIR stacked photodetector and several prevalent existing detectors, which enabled us to summarize the unique advantages of our device. In Table 1, when compared with detectors that use different materials, the response speed of our stacked photodetector shows advantages, being 1.2 and 15.2 times faster than photodetectors based on Si-PbS [39] and Perovskite-Si [20]. Moreover, our detector stands out with its ability to cover a broader detection wavelength range [14]. The detection bands of the other three detectors in the infrared part only extend up to 1600 nm at most, whereas ours can reach around 2800 nm. Overall, our detector exhibits better performance.

## 4. Conclusions

In summary, this paper presents a VIS-SWIR stacked photodetector, which is capable of detecting wavelengths in the range of visible to short-wave infrared at room temperature. The experimental results indicate that HgTe CQD film can pass through part of visible light and show good compatibility with silicon. The vertically stacked structure not only broadens the operating wavelength range but also reduces the area requirement.

Under the light source of a tungsten lamp, the photocurrent of the VIS-SWIR stacked photodetector can reach 112.5 μA, while under the blackbody, it is 1.24 μA. The response speed can achieve 1.65 μs. The specific detectivities of the VIS photodetector and SWIR photodetector are 6.76 × 10^10^ Jones and 1.41 × 10^10^ Jones, respectively. After stacking the VIS and SWIR photodetectors, specific detectivities of the VIS photodetector and SWIR photodetector increase to 1.01 × 10^11^ and 2.66 × 10^10^ Jones. Respectively, specific detectivities increase by 1.5 and 1.8 times. The VIS-SWIR stacked photodetector has the ability to detect light with varying wavelengths and color temperatures, which facilitates its application in aspects such as remote sensing, imaging, and target detection. Additionally, we design a TIA circuit to amplify the tiny current output of this VIS-SWIR stacked photodetector, accompanied by matching regulator and filter modules. The magnification of the circuit is adjusted to 25,000 based on the photocurrents. This circuit is capable of amplifying the current of 120 μA to 3 V, and the maximum amplified voltage is less than the saturation voltage of 3.6 V, ensuring stable and reliable operation.

## Figures and Tables

**Table 1:** Performance comparison of detectors.

Photodetectors	Detectivity (Jones)	Response Speed (μs)	Spectral Range (nm)	Data Source
Si-HgTe	1.01 × 10^11^ (VIS) 2.66 × 10^10^ (SWIR)	1.65	430–2800	This work
Si-PbS	1.47 × 10^11^	2.04	600–1600	[39]
Perovskite-Si	5.56 × 10^13^	25	400–1100	[20]
Si-Ge	1.2 × 10^11^	1–2	400–1550	[14]

All the data were obtained at a temperature of 300 K.

## Data Availability

The data that support the findings of this study are available from the corresponding author upon reasonable request.

## References

[1] Meng L., Xu Q., Zhang J., Wang X. (2024). Colloidal Quantum Dot Materials for Next-Generation near-Infrared Optoelectronics. Chem. Commun..

[2] Zeng L., Chen Q., Zhang Z., Wu D., Yuan H., Li Y., Qarony W., Lau S.P., Luo L., Tsang Y.H. (2019). Multilayered PdSe_2_/Perovskite Schottky Junction for Fast, Self-Powered, Polarization-Sensitive, Broadband Photodetectors, and Image Sensor Application. Adv. Sci..

[3] Sun S., Li Y., Chen F. (2024). Innovative CQD Detector for Broadband Multispectral Imaging. Light Sci. Appl..

[4] Chen P., Wu Z., Shi Y., Li C., Wang J., Yang J., Dong X., Gou J., Wang J., Jiang Y. (2021). High-performance Silicon-based PbSe-CQDs Infrared Photodetector. J. Mater. Sci. Mater. Electron..

[5] Mauthe S., Baumgartner Y., Sousa M., Ding Q., Rossell M.D., Schenk A., Czornomaz L., Moselund K.E. (2020). High-Speed III-V Nanowire Photodetector Monolithically Integrated on Si. Nat. Commun..

[6] Gong W., Gao F., Bai J., Jiang Y., Wang T., Yan J., Li L. (2024). A Design Strategy for Low-Cost Single/Dual-Band Photodetector: Bulk Heterojunction and Interface Engineering. Small.

[7] Wang B. (2022). High-Speed Si-Ge Avalanche Photodiodes. PhotoniX.

[8] Wang F., Wang Z., Xu K., Wang F., Wang Q., Huang Y., Yin L., He J. (2015). Tunable GaTe-MoS_2_ van Der Waals p–n Junctions with Novel Optoelectronic Performance. Nano Lett..

[9] Elahi E., Rabeel M., Ahmed B., Aziz J., Suleman M., Khan M.A., Rehman S., Rehmat A., Asim M., Rehman M.A. (2024). Revealing Bipolar Photoresponse in Multiheterostructured WTe_2_–GaTe/ReSe_2_–WTe_2_ P–N Diode by Hybrid 2D Contact Engineering. ACS Appl. Mater. Interfaces.

[10] Mohammed W.F., Humoody M.A., Al-Tikriti M.N. (2013). Simulation of Photogenerated Current of PN Silicon Photodetector Enhanced by Impurity Photovoltaic Effect. Renew. Sustain. Energy Rev..

[11] Cansizoglu H., Ponizovskaya Devine E., Gao Y., Ghandiparsi S., Yamada T., Elrefaie A.F., Wang S.-Y., Islam M.S. (2017). A New Paradigm in High-Speed and High-Efficiency Silicon Photodiodes for Communication—Part I: Enhancing Photon–Material Interactions via Low-Dimensional Structures. IEEE Trans. Electron Devices.

[12] Xue X., Luo Y., Hao Q., Cao J., Tang X., Liu Y., Chen M. (2023). Low Dark-Current Quantum-Dot Infrared Imager. ACS Photonics.

[13] Liu M., Yazdani N., Yarema M., Jansen M., Wood V., Sargent E.H. (2021). Colloidal Quantum Dot Electronics. Nat. Electron..

[14] Siontas S., Li D., Wang H., Aravind A.V.P.S., Zaslavsky A., Pacifici D. (2019). High-Performance Germanium Quantum Dot Photodetectors in the Visible and near Infrared. Mater. Sci. Semicond. Process..

[15] Kuo M.-H., Lai W.-T., Lee S.-W., Chen Y.-C., Chang C.-W., Chang W.-H., Hsu T.-M., Li P.-W. (2015). Design of Multifold Ge/Si/Ge Composite Quantum-Dot Heterostructures for Visible to near-Infrared Photodetection. Opt. Lett..

[16] Hwang A., Park M., Park Y., Shim Y., Youn S., Lee C.-H., Jeong H.B., Jeong H.Y., Chang J., Lee K. (2021). Visible and Infrared Dual-Band Imaging via Ge/MoS_2_ van Der Waals Heterostructure. Sci. Adv..

[17] Sridhar S.R., Tailor N.K., Satapathi S., Kumar B. (2024). Narrow Dual-Band Photodetector Based on Cs_2_ AgBiBr_6_ Lead-Free Double Perovskite Single Crystal. IEEE Trans. Electron Devices.

[18] Huang B., Liu J., Han Z., Gu Y., Yu D., Xu X., Zou Y. (2020). High-Performance Perovskite Dual-Band Photodetectors for Potential Applications in Visible Light Communication. ACS Appl. Mater. Interfaces.

[19] Kim W., Seo Y., Ahn D., Kim I.S., Balamurugan C., Jung G.Y., Kwon S., Kim H., Pak Y. (2024). Monolithic Perovskite–Silicon Dual-Band Photodetector for Efficient Spectral Light Discrimination. Adv. Sci..

[20] Liu Y., Lin D., Xing J., Zhao H., Wan H., Wang H., Ji Z., Chen X. (2024). Mode-Switching Single-Pixel Imaging via a High-Performance Perovskite-Si Dual-Mode Photodetector. Photonics Res..

[21] Qiu Y., Yan N., Yao H., Chen M. (2023). Plasmon-Enhanced HgTe Colloidal Quantum Dot Infrared Photodetectors. Infrared Phys. Technol..

[22] Xue X., Hao Q., Chen M. (2024). Very Long Wave Infrared Quantum Dot Photodetector up to 18 μm. Light Sci. Appl..

[23] Xia K., Gao X.D., Fei G.T., Xu S.H., Liang Y.F., Qu X.X. (2024). High-Performance Visible to Mid-Infrared Photodetectors Based on HgTe Colloidal Quantum Dots under Room Temperature. ACS Appl. Mater. Interfaces.

[24] Qin Y., Guo T., Liu J., Lin T., Wang J., Chu J. (2023). Colloidal Quantum Dots in Very-Long-Wave Infrared Detection: Progress, Challenges, and Opportunities. ACS Omega.

[25] Hu Z., Qin Y., Liu J., Guo T., Wang J. (2024). Postsynthetic Size Focusing via Digestive Ripening in HgTe Quantum Dots. J. Phys. Chem. Lett..

[26] Xue X., Chen M., Luo Y., Qin T., Tang X., Hao Q. (2023). High-Operating-Temperature Mid-Infrared Photodetectors via Quantum Dot Gradient Homojunction. Light Sci. Appl..

[27] Zhao X., Yao H., Qiu Y., Yan N., Hao Q., Chen M. (2024). Assessing the Potential and Limitations of PbS and HgTe Colloidal Quantum Dot Infrared Detectors for Free Space Optical Communication. Adv. Mater. Technol..

[28] Geisz J.F., France R.M., Schulte K.L., Steiner M.A., Norman A.G., Guthrey H.L., Young M.R., Song T., Moriarty T. (2020). Six-Junction III–V Solar Cells with 47.1% Conversion Efficiency under 143 Suns Concentration. Nat. Energy.

[29] Laoufi A.M., Dennai B., Kadi O., Fillali M. (2021). Numerical Modeling of Multi-Junction Solar Cell-Based CIGS with Two Sub-Cells in Parallel Using Silvaco TCAD. Chalcogenide Lett..

[30] Wang S., Xiang X., Zhou C., Zhai Y., Quan R., Wang M., Hou F., Zhang S., Dong R., Liu T. (2017). Simulation of High SNR Photodetector with L-C Coupling and Transimpedance Amplifier Circuit and Its Verification. Rev. Sci. Instrum..

[31] Lee G., Lee J., Kang C. (2019). Strong and Sustainable Chemical Bonding of TiO_2_ on Nylon Surface Using 3-Mercaptopropyltrimethoxysilane (3-MPTMS): Analysis of Antimicrobial and Decomposition Characteristics of Contaminants. J. Coat. Technol. Res..

[32] Kohler T., Hejtmann G., Henneck S., Schubert M., Guyenot M. (2022). Sol–gel Encapsulation for Power Electronics Utilizing 3-Glycidyloxypropyltriethoxysilane and 3-Mercaptopropyltrimethoxysilane. J. Sol Gel Sci. Technol..

[33] Ackerman M.M., Chen M., Guyot-Sionnest P. (2020). HgTe Colloidal Quantum Dot Photodiodes for Extended Short-Wave Infrared Detection. Appl. Phys. Lett..

[34] Tang X., Ackerman M.M., Chen M., Guyot-Sionnest P. (2019). Dual-Band Infrared Imaging Using Stacked Colloidal Quantum Dot Photodiodes. Nat. Photonics.

[35] Li S.S. (2006). Semiconductor Physical Electronics.

[36] Liu E., Zhu B., Luo J. (2017). The Physics of Semiconductor.

[37] Chen M., Xue X., Qin T., Wen C., Hao Q., Tang X. (2023). Universal Homojunction Design for Colloidal Quantum Dot Infrared Photodetectors. Adv. Mater. Technol..

[38] Chen M., Guyot-Sionnest P. (2017). Reversible Electrochemistry of Mercury Chalcogenide Colloidal Quantum Dot Films. ACS Nano.

[39] Xu K., Xiao X., Zhou W., Jiang X., Wei Q., Chen H., Deng Z., Huang J., Chen B., Ning Z. (2020). Inverted Si:PbS Colloidal Quantum Dot Heterojunction-Based Infrared Photodetector. ACS Appl. Mater. Interfaces.

